# Positive rates of endometrial and cervical cytology in ovarian cancer patients with *BRCA1/2* germline pathogenic variants

**DOI:** 10.1186/s43046-026-00344-z

**Published:** 2026-02-16

**Authors:** Eitaro Funada, Hidetaka Nomura, Tsubasa Kon, Yuri Kuratomi, Toshiaki Watanabe, Mayumi Kamata, Atsushi Fusegi, Akiko Abe, Shogo Nishino, Motoko Kanno, Sachiho Netsu, Makiko Omi, Yoichi Aoki, Terumi Tanigawa, Sanshiro Okamoto, Mayu Yunokawa, Naoko Yamazaki, Tomohiro Chiba, Yui Kojima, Akiko Tonooka, Hiroyuki Kanao

**Affiliations:** Cancer Institute Hospital of Japanese Foundation for Cancer Research, Tokyo, Japan

**Keywords:** Endometrial cytology, Cervical cytology, Positive rate, Ovarian cancer, HBOC, Cancer surveillance

## Abstract

**Background:**

The optimal approach for early-stage ovarian cancer detection remains unclear, particularly in patients with hereditary breast and ovarian cancer (HBOC). As ovarian cancer in HBOC is often thought to originate from the fallopian tube fimbriae, endometrial cytology may play a role in detecting early-stage disease. This retrospective study aimed to assess the positive rate of endometrial cytology and compare to cervical cytology in ovarian cancer patients with patients with BRCA1/2 germline pathogenic variants (gPV).

**Methods:**

Patients with genetically confirmed *BRCA1/2* gPV diagnosed with ovarian, fallopian tube, or primary peritoneal cancer from May 1998 to April 2024 at our institution were included. Those who underwent endometrial and cervical cytology prior to initial treatment were eligible for this analysis. The positive rates for each cytology type were evaluated.

**Results:**

A total of 154 patients with *BRCA1/2* gPV were enrolled, 118 underwent both endometrial and cervical cytology. The positive rate for endometrial cytology was significantly higher than that for cervical cytology (36.4% vs. 15.3%; *p* < 0.001). Additionally, a correlation was observed between the positive rate of endometrial cytology and cancer stage (Stage I 16.6%, Stage II 25%, Stage III 41.5%, Stage IV 58.6%; *r* = 0.9895).

**Conclusion:**

In this retrospective study, the positive rate of endometrial cytology surpassed that of cervical cytology. Endometrial cytology may play a role as a potential surveillance tool for women with *BRCA1/2* gPV, even at early stages of ovarian cancer.

## Background

Hereditary breast and ovarian cancer (HBOC) is associated with germline pathogenic variants (gPV) in *BRCA1/2*, which increase the risk of several cancers, including breast and ovarian cancer. Risk-reducing bilateral salpingo-oophorectomy (RRSO) is a well-established method for lowering the risk of ovarian cancer in women with *BRCA1/2* gPV [1]. However, effective surveillance strategies to reduce ovarian cancer mortality rate in these patients, as an alternative to RRSO, remain unclear. A previous study in the UK found that screening for ovarian cancer using serum CA-125 and transvaginal ultrasound did not significantly reduce mortality rates [2]. Other studies have examined high-risk populations, including those with *BRCA1/2* gPV, and demonstrated that screening can effectively detect early-stage cancers, but it remains uncertain whether it improves survival outcomes [3, 4].

In Japan, endometrial cytology is commonly used as a screening tool for endometrial cancer. However, in some cases of ovarian cancer, malignant cells may be identified through endometrial cytology due to their origin in the fallopian tubes. We previously reported a case of serous tubal intraepithelial carcinoma (STIC) diagnosed via endometrial cytology [5], suggesting that this method could be useful for surveillance in women with *BRCA1/2* gPV to detect early-stage ovarian cancer. In fact, we identified five cases of ovarian cancer through our surveillance program that included endometrial cytology. These cases were previously reported in detail [6], and they support the utility of this approach in early detection.

A systematic review reported that the positive rate of endometrial cytology for ovarian, fallopian tube, and primary peritoneal cancer is 23% [7]. However, this study focused solely on patients with *BRCA1/2* gPV, whose cancers are believed to originate from STIC [8]. While cervical cytology may also detect ovarian cancer [9–11], the comparison between endometrial and cervical cytology for this purpose is still unclear.

We hypothesized that endometrial or cervical cytology could be beneficial for routine surveillance in women with *BRCA1/2* gPV if their sensitivity is higher in ovarian cancer patients with these variants. The primary objective of this study was to determine the positive rates of endometrial and cervical cytology in ovarian cancer patients with *BRCA1/2* gPV, conducting a retrospective analysis to address these clinical questions.

## Methods

### Study design and population

Following approval from the Institutional Review Board, a single-center retrospective observational study was conducted at the Cancer Institute Hospital of the Japanese Foundation for Cancer Research in Tokyo, Japan, with participants enrolled from May 1998 to April 2024. Patients were genetically diagnosed with *BRCA1/2* gPV. Among these patients, 154 cases of ovarian, fallopian tube, and primary peritoneal cancer were identified, and 118 patients who underwent both endometrial and cervical cytology before treatment as part of their initial consultation before any treatment were included in this study. These cytologic assessments were performed as part of routine cancer surveillance in high-risk individuals or preoperative work-up.

### Materials

For cervicovaginal cytology, we used a disposable plastic brush (J Fit Brush, Muto Pure Chemicals Co., Ltd.) or a cotton swab, while a disposable nylon brush (Honest Keikan Brush, Honest Medical Co., Ltd.) was employed for cervical canal cytology. For endometrial cytology, a different disposable nylon brush (Honest Super Brush, Honest Medical Co., Ltd.) was utilized. Samples were smeared onto slides, fixed with ethanol, and then diagnosed using the Papanicolaou smear technique. Results were considered positive if the pathological findings included atypical glandular cells (AGC), adenocarcinoma in situ, adenocarcinoma, or other malignant neoplasms in cervical cytology and if they were positive or suspicious for malignancy in endometrial cytology. We then calculated the positive rate for each type of cytology.

### Statistical analysis

Continuous variables were expressed as medians and compared using the Mann–Whitney U test. Categorical variables were analyzed using Fisher’s exact test. The statistical analysis was conducted with R software, version 3.0.1 (The R Foundation for Statistical Computing, Vienna, Austria).

## Results

Of the 154 women with *BRCA1/2* gPV who developed ovarian, fallopian tube, and primary peritoneal cancer, 118 underwent endometrial and cervical cytology before treatment. Table 1 presents the characteristics of these patients, with a median age of 49 years (range: 32–81). The majority of the histological cancer types were high-grade serous carcinoma (HGSC), which accounted for 93.2% of cases. All cancer stages were represented, but nearly 80% of the patients had advanced cancer (Stage Ⅲ and Stage Ⅳ).

**Table 1:** Characteristics of patients (*n* = 118)

Age, years (range)	49 (32–81)
Histological type	
HGSC	110 (93.2%)
Endometrioid carcinoma	4 (3.4%)
Clear cell carcinoma	1 (0.8%)
Mucinous carcinoma	1 (0.8%)
Carcinosarcoma	1 (0.8%)
Mixed carcinoma	1 (0.8%)
FIGO^*^ stage	
Stage I	12 (10.2%)
Stage II	12 (10.2%)
Stage III	65 (55.1%)
Stage IV	29 (24.6%)

^*^International Federation of Gynecology and Obstetrics (FIGO)

Table 2 displays the positive rates of cervical and endometrial cytology for each stage of ovarian cancer in patients with *BRCA1/2* GPVs. The positive rate of abnormalities was significantly higher for endometrial cytology compared to cervical cytology (36.4% vs. 15.3%; *p* < 0.001). A linear relationship was observed between cancer stage and the positive rate of abnormalities, with higher rates associated with more advanced cancer stages.

**Table 2:** Positive rates of malignant cells for each type of cytology in *BRCA1/2*-related ovarian cancer patients

Number of positive patients (positive rate)		
FIGO^*^ stage	Cervical cytology	Endometrial cytology
Stage I (12 cases)	1 (8.3%)	2 (16.6%)
Stage II (12 cases)	2 (16.7%)	3 (25%)
Stage III (65 cases)	8 (12.3%)	27 (41.5%)
Stage IV (29 cases)	7 (24.1%)	17 (58.6%)
Total (118 cases)	18 (15.3%)	43 (36.4%)

^*^International Federation of Gynecology and Obstetrics (FIGO)

**Fig. 1:**
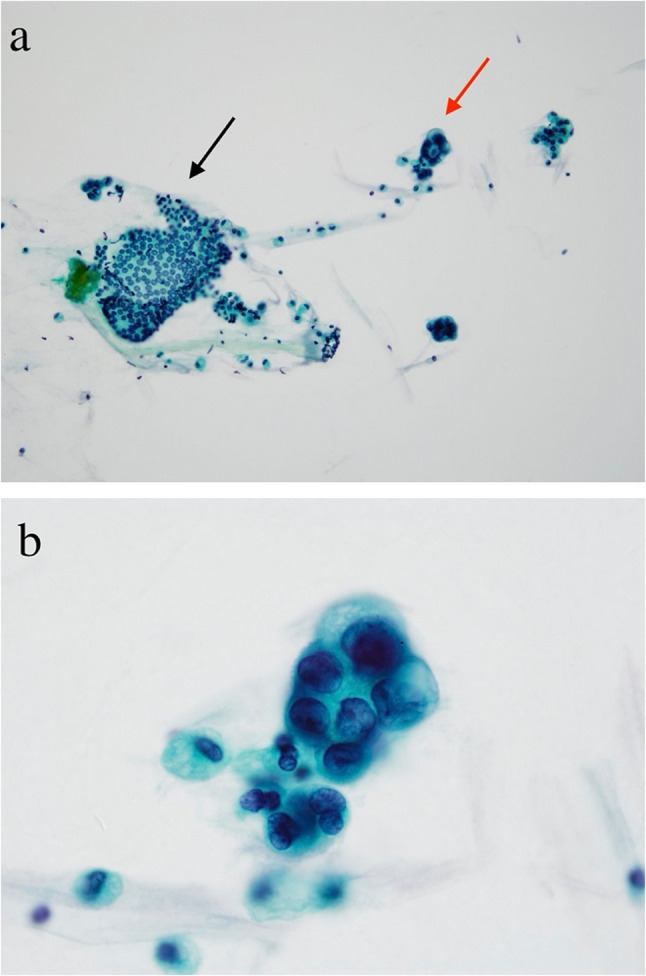
Endometrial cytology of Stage I *BRCA1/2-*related ovarian cancer. Papanicolaou smear: (**a**, ×20 magnification; **b**, ×100 magnification)

We identified two Stage Ⅰ ovarian cancer patients with *BRCA1/2* gPV who had positive endometrial cytology, and their characteristics are presented in Table 3. One patient (case 1) had synchronous double cancer of the ovary and endometrium, leading to the detection of malignant cells in the endometrial cytology, suggesting endometrial carcinoma. In contrast, the other patient (case 2) exhibited abnormalities in both endometrial and cervical cytology, but her tumor markers were not elevated, and no abnormal lesions were found on magnetic resonance imaging (MRI) or computed tomography (CT) scans. This patient underwent total laparoscopic hysterectomy and bilateral salpingo-oophorectomy, and the pathological results indicated that the histological cancer type was HGSC, with the primary site in the right fallopian tube and a tumor size of only 1 × 1 × 5 mm. This case demonstrates that early-stage ovarian cancer can be detected through cytology, which was not identified by other examinations. Figure 1a and b illustrate the cytology of Stage I ovarian cancer, where small clusters of atypical cells were observed against a clean background of atrophic endometrium and histiocytes. The atypical cells exhibited a high nuclear-to-cytoplasmic ratio, nuclear enlargement, irregular nuclear contours, and increased chromatin. Based on their appearance, these findings suggest the possibility of extrauterine malignancy, such as ovarian or fallopian tube cancer.

**Table 3:** Characteristics of stage I *BRCA1/2*-related ovarian cancer cases with positive endometrial cytology

Case	1	2
Endometrial cytology	Positive (Endometrial carcinoma suspected)	Positive (extrauterine malignant tumor suspected)
Cervical cytology	NILM^*^	AGC^*^
Serum CA125 (U/ml)	519	10.8
MRI	Right ovarian cancer	No evidence of disease
PET-CT	Hot spot at right ovary	No hot spot
Surgical procedure	TAH + BSO + OM + PLA + PALA^†^	TLH + BSO^†^
Primary focus	Right ovary	Right fallopian tube
Tumor size of ovary (mm)	85 × 55 × 25	1 × 1 × 5
Histological cancer type	HGSC^‡^	HGSC^‡^
STIC	Positive	Positive
Cytology of peritoneal washing	Positive	Negative

*NILM: negative for intraepithelial lesion or malignancy; AGC: atypical glandular cells

†TAH: total abdominal hysterectomy; BSO: bilateral salpingo-oophorectomy; OM: omentectomy; PLA: pelvic lymphadenectomy; PALA: para-aortic lymphadenectomy; TLH: total laparoscopic hysterectomy

‡HGSC: high-grade serous carcinoma

## Discussion

To the best of our knowledge, this is the first article to report the positive rates of endometrial and cervical cytology in ovarian cancer patients with *BRCA1/2* gPV, showing that endometrial cytology had a significantly higher positive rate than cervical cytology. We also discussed the relationship between the positive rates of cytology and cancer stage, noting that the positive rates increased with more advanced cancer stage.

A recent systematic review indicated that the positive rate of endometrial cytology for ovarian, fallopian tube, and primary peritoneal cancer was 23%. It also found that aspiration smear sampling for endometrial cytology had a higher positive rate than brush smear sampling (33% vs. 13%) [7]. Although we utilized brush smears, which are expected to yield a lower positive rate, our observed rate was 36.4%, exceeding that of previous studies. Our report focused on ovarian cancer patients with *BRCA1/2* gPV, particularly HGSC, which is primarily believed to originate from STIC [12], malignant cells may shed into the endometrial cavity, thus increasing the likelihood of detection by endometrial cytology. In a previous study, we reported a 50% positive rate of endometrial cytology in fallopian tube cancer cases [13]. Additionally, the tubal fimbriae are now considered the primary malignant region for ovarian cancer patients with *BRCA1/2* gPV [14]. Consequently, the positive rate of endometrial cytology for ovarian cancer patients with *BRCA1/2* gPV was higher than in previous studies that included all ovarian cancer cases.

Patients undergoing endometrial cytology often experience pain, and a previous systematic review indicated that lidocaine is an effective medication for pain relief [15]. In contrast, cervical cytology, commonly used for screening uterine cervical cancer, typically does not require medication for the examination. There is a report that identified STIC from cervical cytology abnormalities [11]. If there is no significant difference in positive rates between endometrial and cervical cytology, cervical cytology would be a reasonable option for detecting ovarian cancer due to its painless and convenient nature compared to endometrial cytology. However, this study found that the positive rate of endometrial cytology was significantly higher than that of cervical cytology in ovarian cancer patients with *BRCA1/2* gPV, suggesting that endometrial cytology could serve as a valuable surveillance tool for women with *BRCA1/2* gPV.

We identified STIC from endometrial cytology [5], which led us to use endometrial cytology for surveillance in women with *BRCA1/2* gPV at our hospital. Through this surveillance, we detected two cases of advanced ovarian cancer with positive endometrial cytology [10]. However, in one instance of Stage I ovarian cancer, there were abnormalities only in the endometrial cytology, while tumor markers and imaging examinations (such as MRI, CT, or positron emission tomography-computed tomography [PET-CT]) showed no abnormalities. This suggests that performing endometrial cytology as part of surveillance for HBOC could potentially detect early-stage ovarian cancer. We found a correlation between the positive rate of endometrial cytology and cancer stage (Stage I 16.6%, Stage II 25%, Stage III 41.5%, and Stage IV 58.6%) (*r* = 0.9895). As cancer stage advances, the likelihood of detecting abnormalities in endometrial cytology increases; the positive rate of endometrial cytology was consistently higher than that of cervical cytology at each cancer stage.

In our study, we included all ovarian cancer patients with *BRCA1/2* gPV. However, we encountered a case where the *BRCA1/2* gPV was not the driver oncogene for the ovarian cancer. This case was classified as a mucinous carcinoma. Through whole-genome sequencing of a sample from a recurrent lesion, we identified another somatic oncogene. Since mucinous carcinoma originates from the ovaries, it is unlikely that preoperative endometrial cytology would yield positive results. This suggests that the positive rate of endometrial cytology could be higher if the focus is strictly on HGSC confirmed *BRCA1/2* gPV, who are more likely to develop from the fimbria of the fallopian tube.

While our findings demonstrate promising sensitivity of endometrial cytology in this population, we acknowledge that our study does not assess the detection rate in a healthy or surveillance population. Thus, the results cannot be extrapolated to screening performance. Further prospective studies are necessary to establish sensitivity, specificity, and cost-effectiss.

## Conclusion

Our analysis indicates that the positive rate of endometrial cytology in ovarian cancer patients with *BRCA1/2* gPV is higher than in previous studies that included all ovarian cancer cases. The positive rate increases with advancing cancer stage. Moreover, we were able to identify abnormalities in endometrial cytology even in Stage I cases. Therefore, endometrial cytology may be a promising component of future surveillance strategies for BRCA1/2 mutation carriers, particularly if supported by further prospective research.

## Data Availability

The data supporting the findings of this study can be obtained from the corresponding author, Hidetaka Nomura, upon reasonable request.

## Abbreviations

HBOC: Hereditary breast and ovarian cancer
gPV: Germline pathogenic variants
RRSO: Risk-reducing bilateral salpingo-oophorectomy
STIC: Serous tubal intraepithelial carcinoma
IRB: Institutional review board
AGC: Atypical glandular cells
HGSC: High-grade serous carcinoma
MRI: Magnetic resonance imaging
CT: Computed tomography
